# Supplementary material for: Improving the Luminescence and Stability of Carbon-Centered Radicals by Kinetic Isotope Effect
Source: Molecules. 2023 Jun 16;28(12):4805. doi: 10.3390/molecules28124805 (PMC10301369; doi:10.3390/molecules28124805)
Supplement: Supplementary file 1 [file molecules-28-04805-s001.zip › molecules-2443127-supplementary.pdf]

*Supplementary Materials*

# Improving the Luminescence and Stability of Radicals by Kinetic Isotope Effect

**Zhichao Ma [1,†], Lintao Zhang [1,†], Zhiyuan Cui [2] and Xin Ai [1,*]**

[1] School of Materials Science and Engineering, Collaborative Innovation Center of Information Technology, Collaborative Innovation Center of Marine Science and Technology, Hainan University, No 58, Renmin Avenue, Haikou 570228, China; 20080500210020@hainanu.edu.cn (Z.M.); zhanglintao@hainanu.edu.cn (L.Z.)
[2] State Key Laboratory of Supramolecular Structure and Materials, College of Chemistry, Jilin University, No. 2699, Qianjin Avenue, Changchun 130012, China; zycui2021@163.com
* Correspondence: aixin133@hainanu.edu.cn
† These authors contributed equally to this work.

**Contents**

**Figure S1** The synthetic routes of deuterated radicals......3
**Figure S2** Mass spectrometry of deuterated radicals. ...... 4
**Figure S3** Infrared absorption (IR) spectrogram of deuterated radicals. ...... 5
**Figure S4** EPR of deuterated radicals in cyclohexane solution at room temperature. ... ...... 5
**Figure S5** UV-Vis absorption spectra of deuterated radicals in solutions of different polarities ($10^{-5}$ M). ...... 6
**Figure S6** Transient fluorescence decay of deuterated radicals in cyclohexane solution ($10^{-5}$ M). ...... 6
**Figure S7** Voltammetry (CV) curves of Non-deuterated radicals. ...... 7
**Figure S8** Voltammetry (CV) curves of deuterated radicals for multiple (20-turn) cycles. ...... 7
**Figure S9** TGA curve of deuterated radicals and Non-deuterated radicals. ...... 8
**Figure S10** Photostability of deuterated radicals and Non-deuterated radicals. ......8
**Table S1** Redox potentials and corresponding orbital energy levels of deuterated radicals and Non-deuterated radicals. ......9
**Table S2** The corresponding SOMO orbital energy levels calculated theoretically and measured experimentally. ......9
**Table S3** Parameters corresponding to the emission bands in TD-DFT calculations of deuterated radicals. ......9

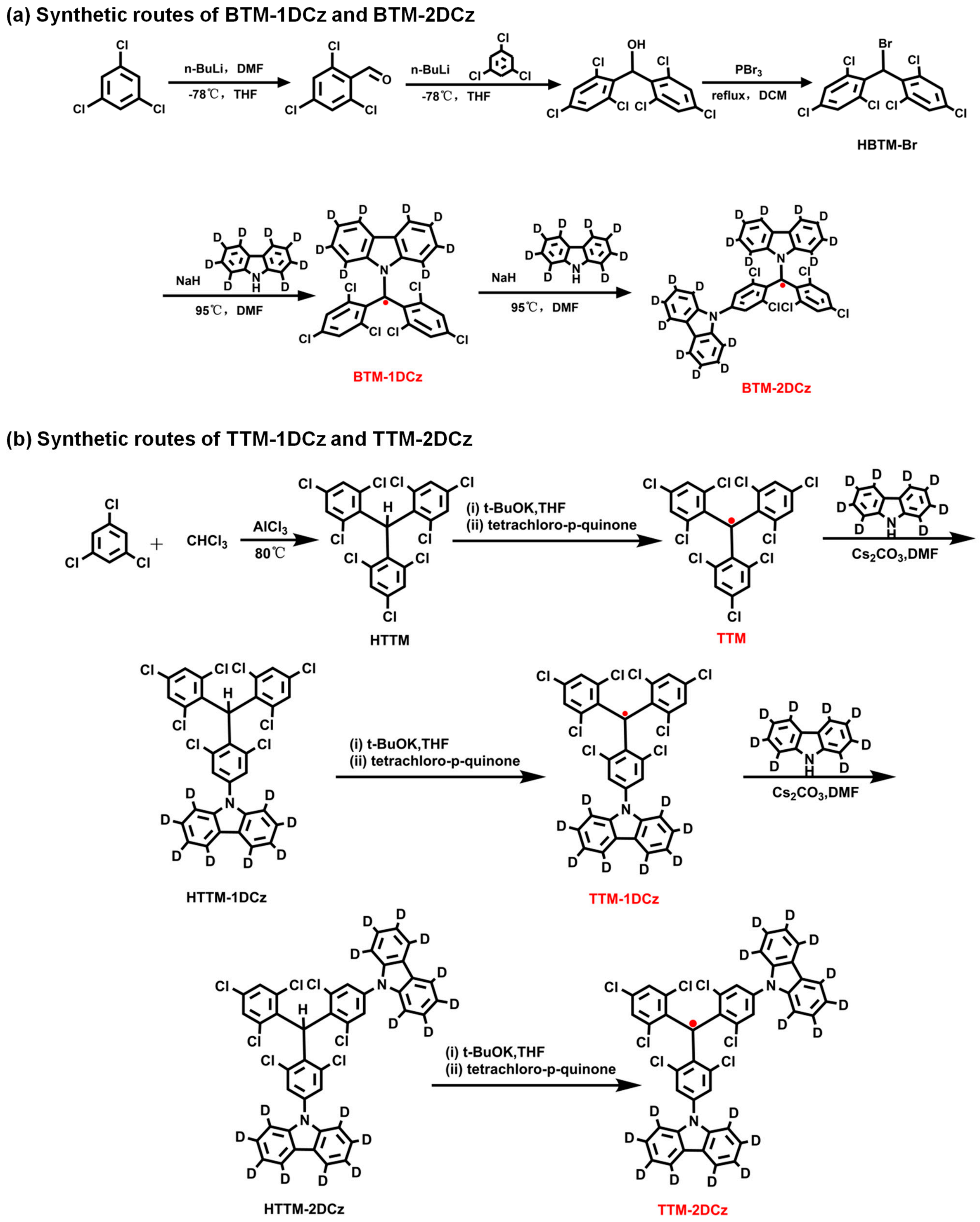


**Figure S1** The synthetic routes of deuterated radicals.

BTM-1DCz LC-HRMS (m/z) calculated for $C_{25}H_4D_8Cl_6N^{\bullet}$ [M]+: 545.9574. Found: 545.9514.

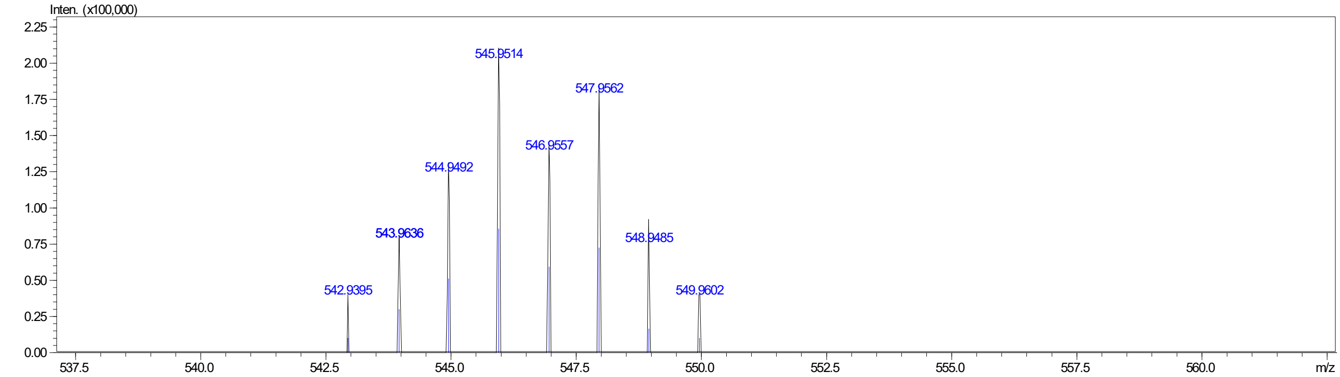


BTM-2DCz LC-HRMS (m/z) calculated for $C_{37}H_4D_{16}Cl_5N_2^{\bullet}$ $[M]^+$: 685.1044. Found: 685.1006.

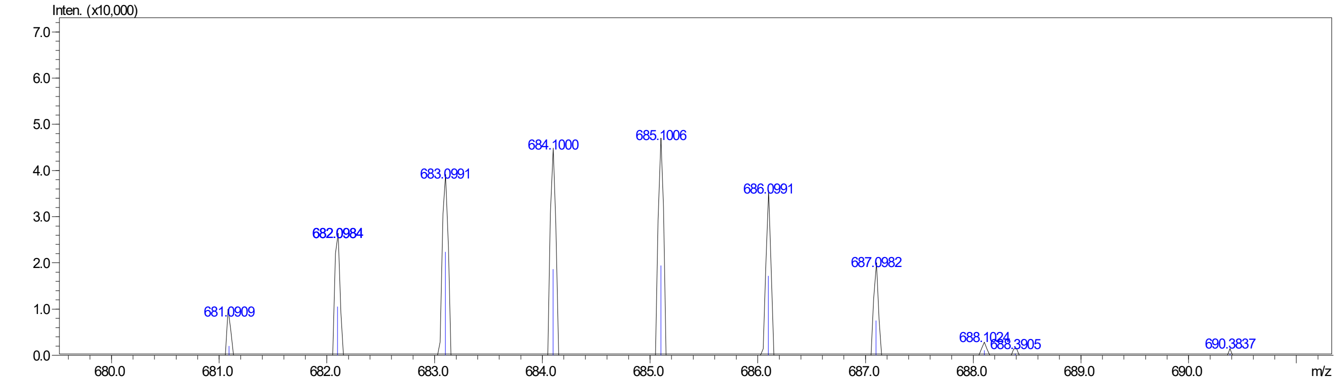


TTM-1DCz LC-HRMS (m/z) calculated for $C_{31}H_6D_8Cl_8N^{\bullet}$ $[M]^+$: 691.9078. Found: 691.9042.

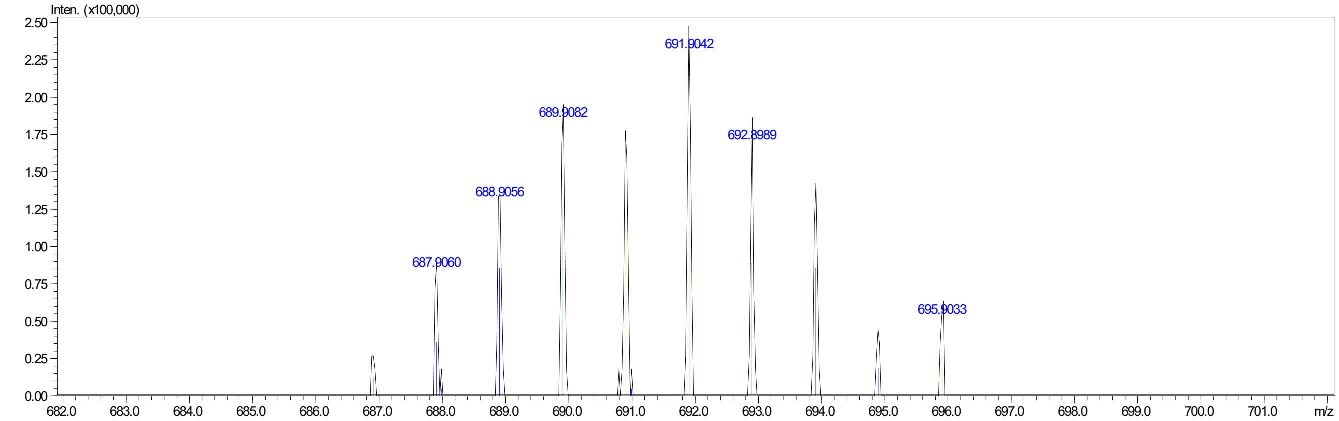


TTM-2DCz LC-HRMS (m/z) calculated for $C_{43}H_6D_{16}Cl_7N_2^{\bullet}$ $[M]^+$ : 829.0517. Found: 829.0498.

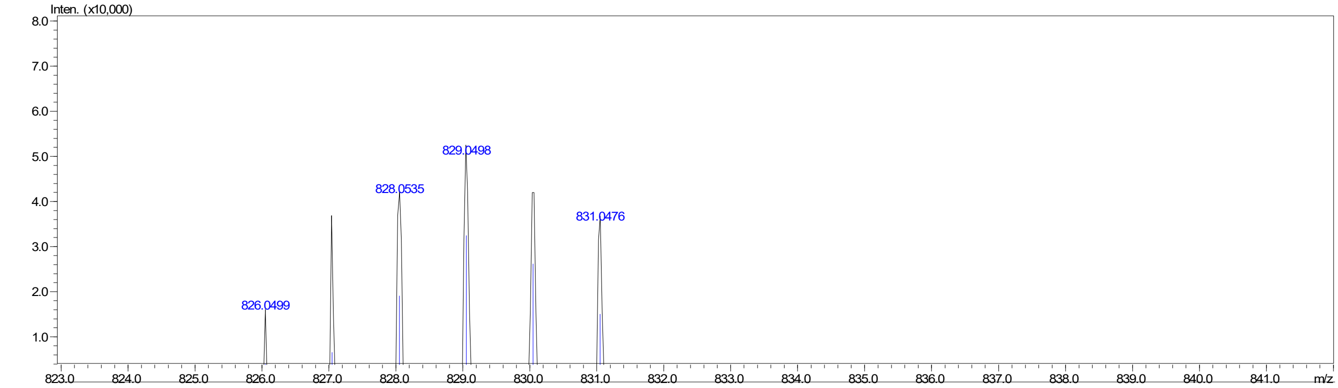


**Figure S2** Mass spectrometry of deuterated radicals.

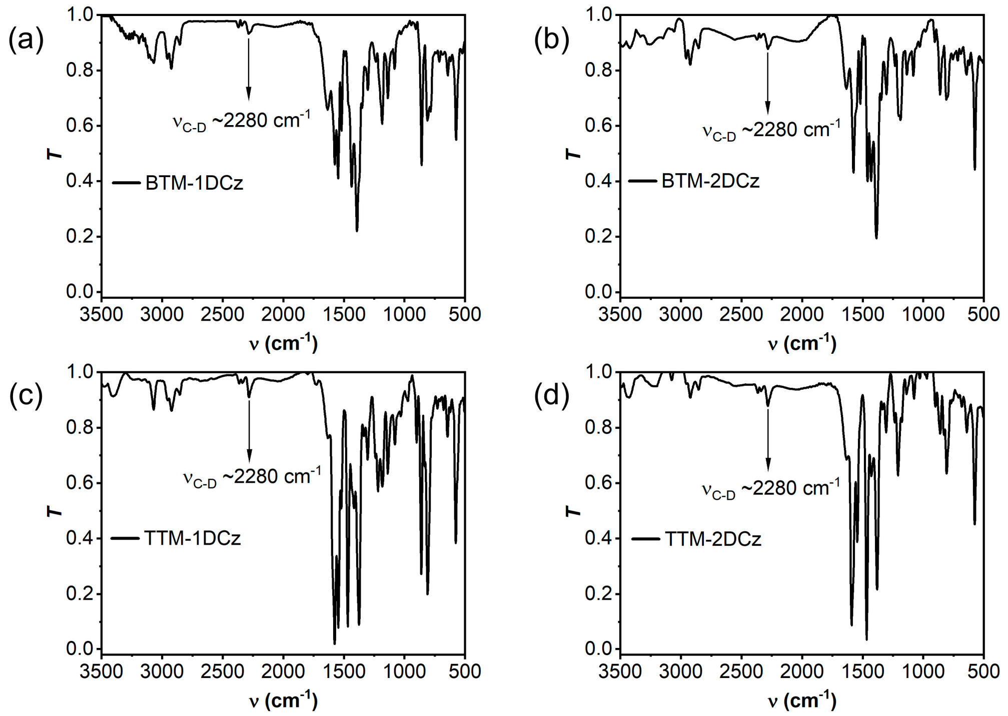


**Figure S3** Infrared absorption (IR) spectrogram of deuterated radicals.

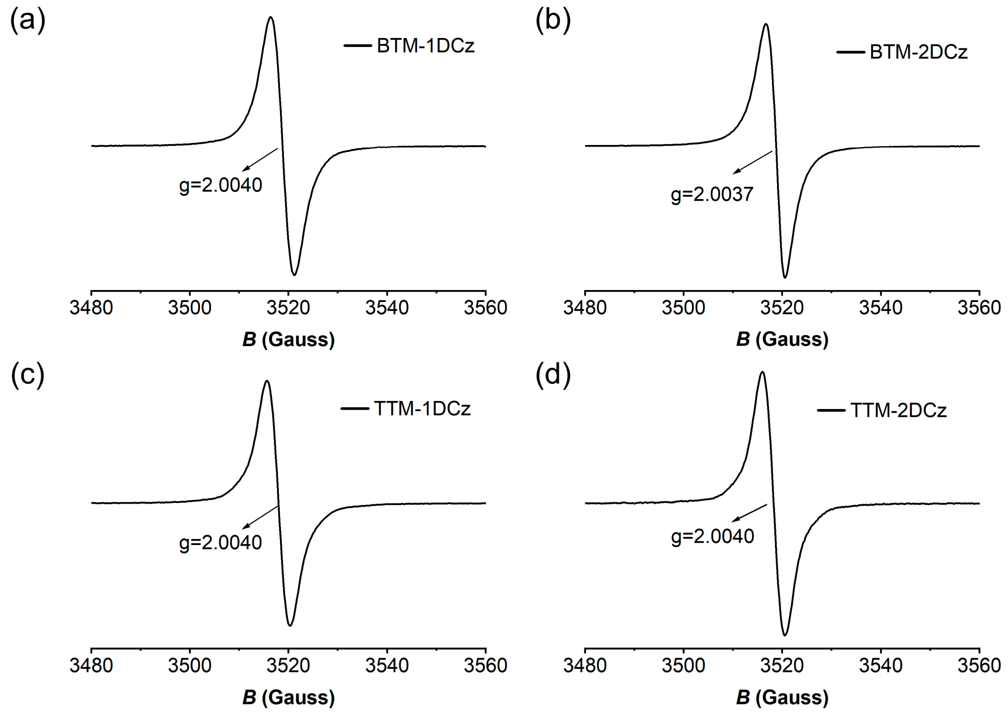


**Figure S4** EPR of deuterated radicals in cyclohexane solution at room temperature.

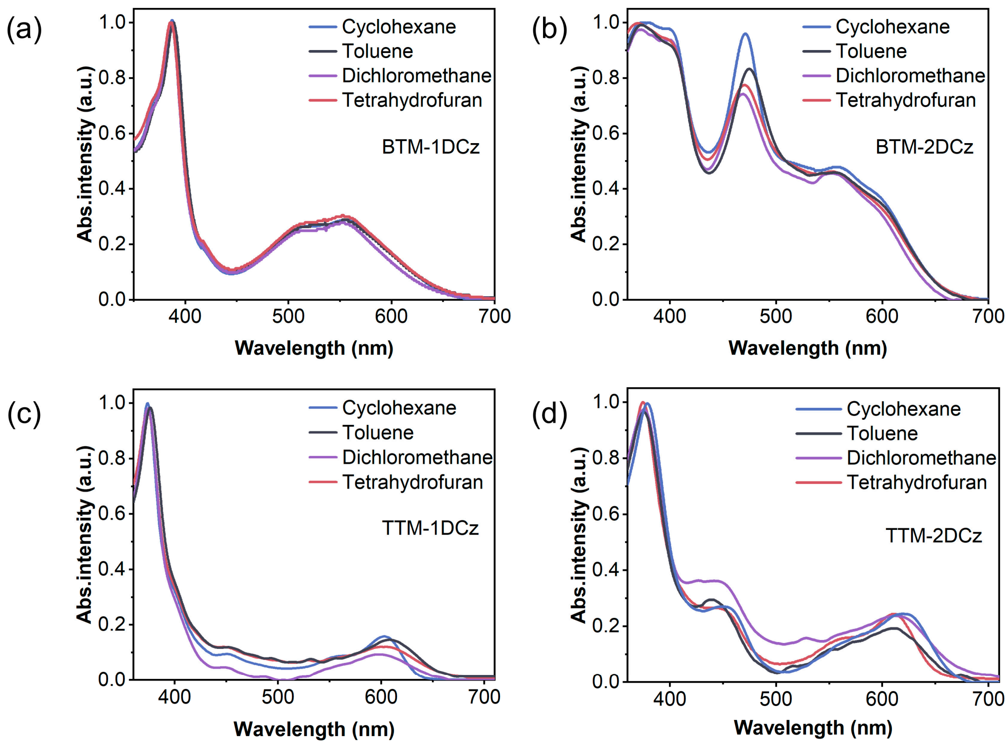


**Figure S5** UV-Vis absorption spectra of deuterated radicals in solutions of different polarities ($10^{-5}$ M).

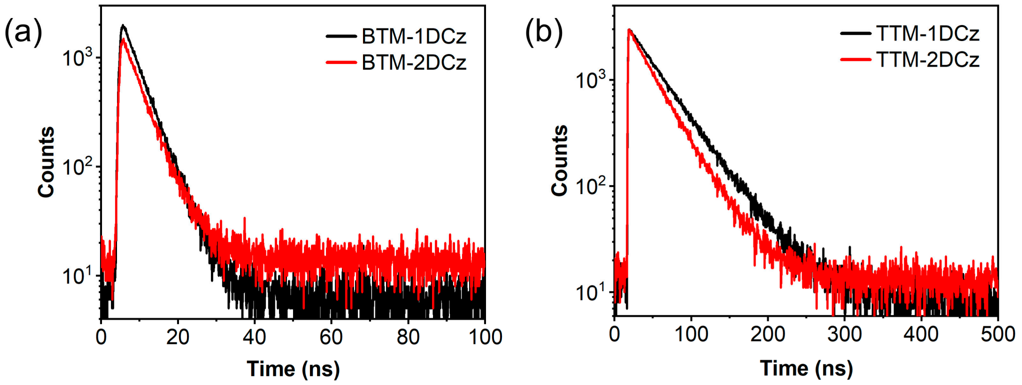


**Figure S6** Transient fluorescence decay of deuterated radicals in cyclohexane solution ($10^{-5}$ M).

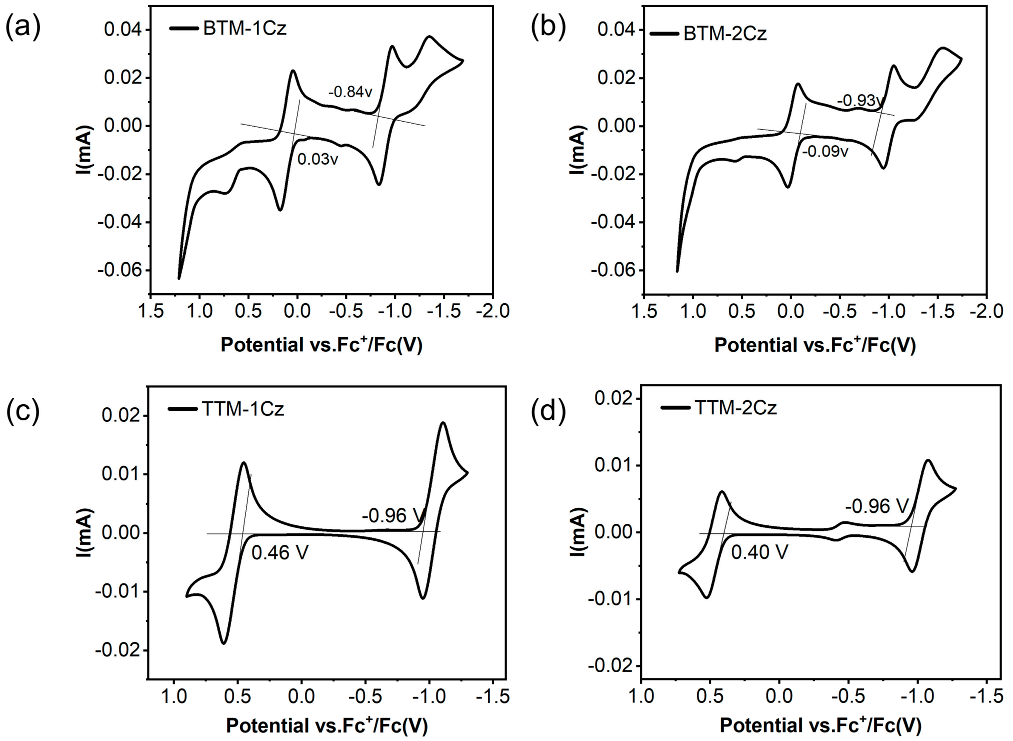


**Figure S7** Voltammetry (CV) curves of Non-deuterated radicals.

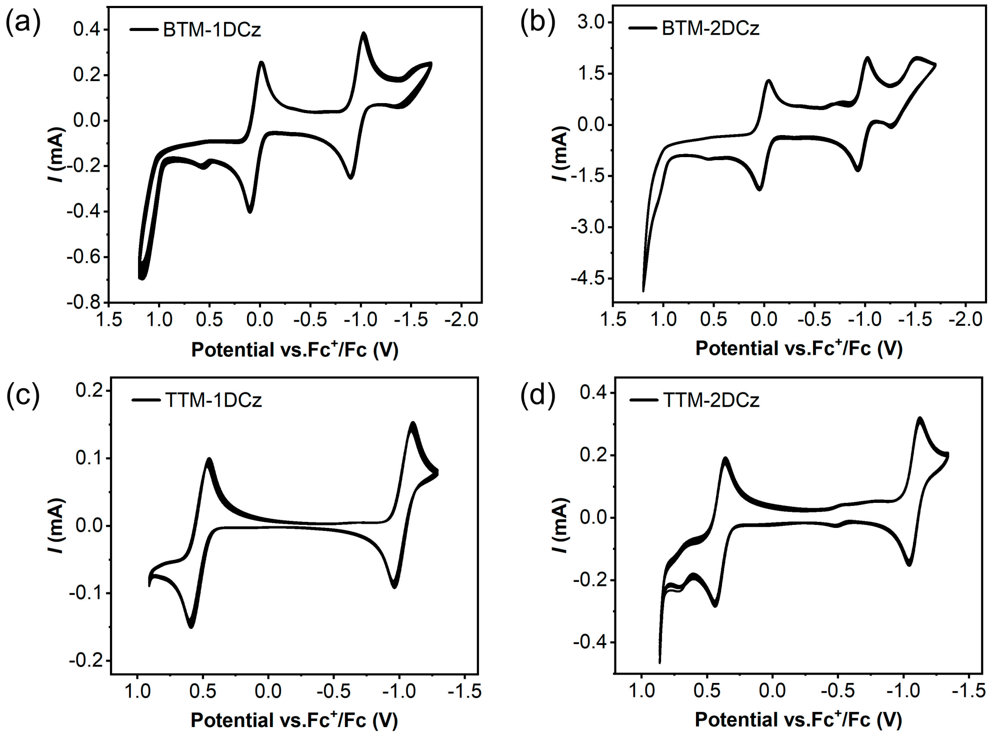


**Figure S8** Voltammetry (CV) curves of deuterated radicals for multiple (20-turn) cycles.

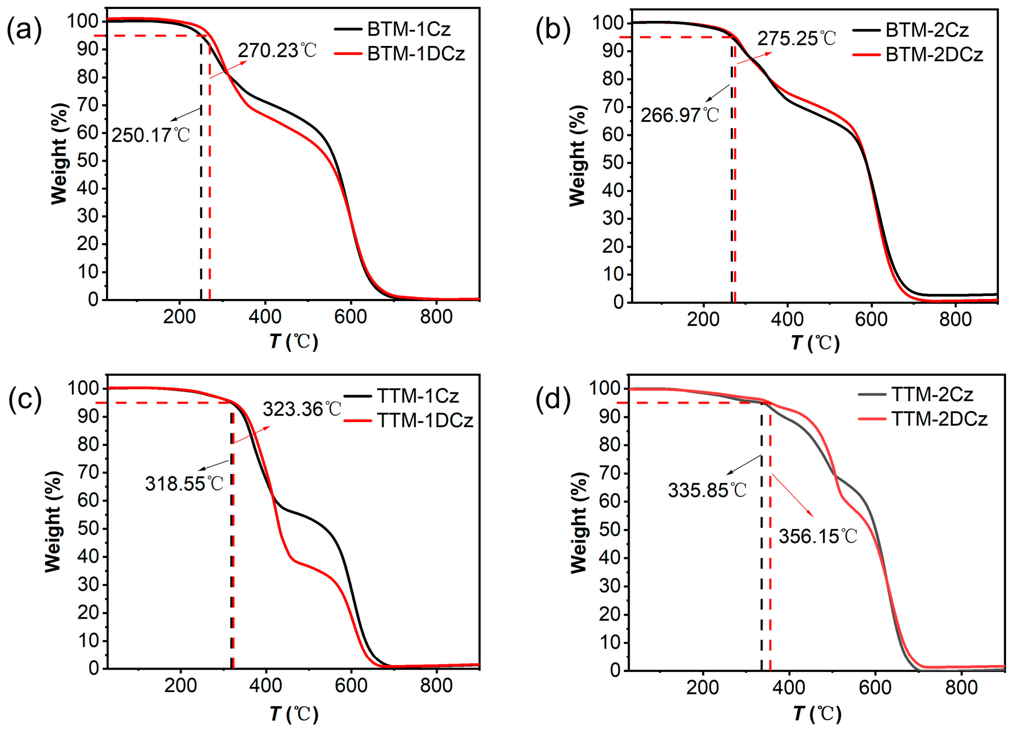


**Figure S9** TGA curve of deuterated radicals and Non-deuterated radicals.

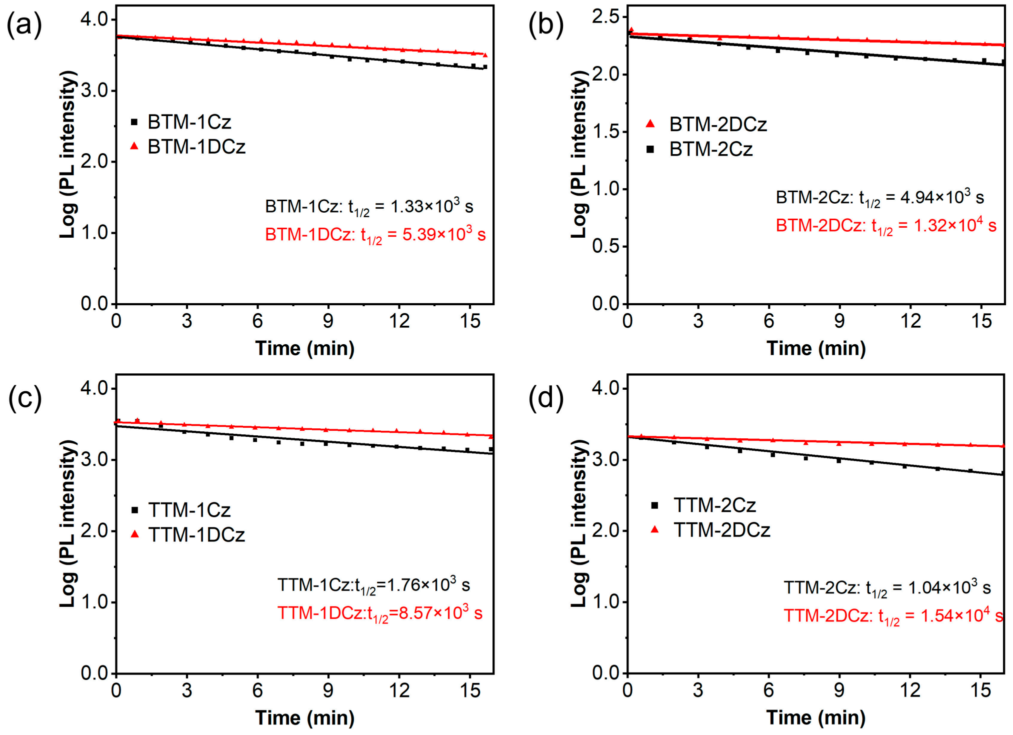


**Figure S10** Photostability of deuterated radicals and Non-deuterated radicals.

**Table S1** Redox potentials and corresponding orbital energy levels of deuterated radicals and non-deuterated radicals.

| | $E_{red}$ (V) | $E_{ox}$ (V) | $E_{\alpha\text{-SOMO}}$ (eV) | $E_{\beta\text{-SUMO}}$ (eV) |
|---|---|---|---|---|
| BTM-1DCz | −0.89 | −0.02 | −4.71 | −3.84 |
| BTM-1Cz | −0.84 | −0.03 | −4.70 | −3.89 |
| BTM-2DCz | −0.92 | −0.08 | −4.65 | −3.81 |
| BTM-2Cz | −0.93 | −0.09 | −4.64 | −3.80 |
| TTM-1DCz | −0.93 | 0.49 | −5.22 | −3.80 |
| TTM-1Cz | −0.96 | 0.46 | −5.19 | −3.77 |
| TTM-2DCz | −0.97 | 0.38 | −5.11 | −3.76 |
| TTM-2Cz | −0.96 | 0.40 | −5.13 | −3.77 |

**Table S2** The corresponding SOMO orbital energy levels calculated theoretically and measured experimentally.

| | SOMO (eV) | | SUMO (eV) | |
|---|---|---|---|---|
| | Calculated | Experimental | Calculated | Experimental |
| BTM-1DCz | −4.99 | −4.71 | −3.01 | −3.84 |
| BTM-2DCz | −4.89 | −4.65 | −2.96 | −3.81 |
| TTM-1DCz | −5.45 | −5.22 | −3.37 | −3.80 |
| TTM-2DCz | −5.33 | −5.11 | −3.30 | −3.76 |

**Table S3** Parameters corresponding to the emission bands in TD-DFT calculations of deuterated radicals.

| Radical | $\lambda_{Em}$ (nm) | orbital excitation contribution | *f* |
|---|---|---|---|
| **BTM-1DCz** | **630** | 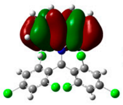 **135 β→136 β (0.94)** 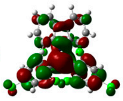 | **0.04** |
| **BTM-2DCz** | **623** | 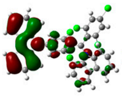 **170 β→171 β (0.80)** 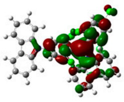 | **0.05** |
| **TTM-1DCz** | **524** | 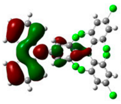 **169 β→172 β (0.24)**<br>**171 β→172 β (0.47)** 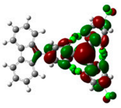 | **0.07** |
| **TTM-2DCz** | **516** | 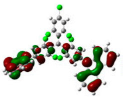 **206 β→207 β (0.48)** 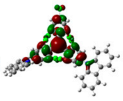 | **0.10** |
